# Risk Factors for Progression of Barrett’s Esophagus to High Grade Dysplasia and Esophageal Adenocarcinoma

**DOI:** 10.1038/s41598-020-61874-7

**Published:** 2020-03-17

**Authors:** Swetha Kambhampati Thiruvengadam, Alan H. Tieu, Brandon Luber, Hao Wang, Stephen J. Meltzer

**Affiliations:** 1 Department of Medicine, Johns Hopkins University School of Medicine, Baltimore, MD United States; 2 Division of Bioinformatics and Biostatistics, Department of Oncology, Johns Hopkins University School of Medicine, Baltimore, MD United States; 3 Division of Gastroenterology and Hepatology, Department of Medicine, Johns Hopkins University School of Medicine, Baltimore, MD United States; 4 Sidney Kimmel Comprehensive Cancer Center, Johns Hopkins University School of Medicine, Baltimore, MD United States; 5 Division of Gastroenterology and Hepatology, Department of Medicine, Eastern Virginia Medical School, Norfolk, VA United States

**Keywords:** Cancer, Oesophageal cancer, Barrett oesophagus

## Abstract

Barrett’s esophagus (BE) is the only known precursor to esophageal adenocarcinoma (EAC). Methods of identifying BE patients at high risk for progression to high-grade dysplasia (HGD) or EAC are needed to improve outcomes and identify who will benefit most from intensive surveillance or ablative therapy. Clinical predictors of BE progression to HGD or EAC are poorly understood, with multiple contradictory studies. We performed a retrospective study which included 460 patients at Johns Hopkins Hospital who underwent at least 2 upper endoscopies 6 months apart showing biopsy-documented BE between 1992 and 2013. Patients with EAC or HGD at the initial endoscopy were excluded. Demographic, clinicopathological, and endoscopic data were collected. Univariate and multivariate Cox proportional hazards analyses with time to progression to HGD and EAC were performed. Among 460 patients included in the study, 132 BE patients developed HGD and 62 developed EAC. Significant EAC risk factors included age, abdominal obesity, caffeine intake, and the presence of HGD. Risk factors for HGD or EAC included age, caffeine intake, and low-grade dysplasia while colonic adenomas trended towards significance. Notably, a history of statin or SSRI usage reduced the risk of EAC or HGD by 49% or 61%, respectively. Our study validated several known and identified several novel risk factors, including a history of colonic adenomas or caffeine usage. Low-grade dysplasia was a risk factor for progression but various endoscopic characteristics were not, suggesting that screening strategies should focus on histology instead. We identified SSRIs as a new potentially chemoprotective medication.

## Introduction

The incidence of esophageal adenocarcinoma (EAC) has increased rapidly in the USA and other nations, but unfortunately, most cases are detected very late, with a fatality rate of 90%^1^. Barrett’s esophagus (BE), the only known precursor for EAC, can progress to low-grade dysplasia (LGD), high-grade dysplasia (HGD), or ultimately EAC. BE is defined as salmon-colored mucosa extending ≥1 cm proximal to the gastroesophageal (GE) junction, with biopsy confirmation of replacement of normal squamous epithelium by metaplastic intestinal-type columnar epithelium^1^. Endoscopic surveillance is currently accepted for all patients with BE, as BE surveillance carries an improved prognosis^2^. Although BE patients have an eleven-fold higher risk of EAC than the general population, their annual risk of this malignancy is 0.11%^3^. These observations have generated controversy regarding cancer screening and surveillance practices. Since most BE patients will not develop EAC, and given the risk and expense of endoscopic surveillance, understanding risk factors for BE progression is important to effectively focus resources on high-risk BE patients, allowing patient stratification and enabling tailored surveillance and therapy.

Predictors of neoplastic progression in BE are incompletely understood. Epidemiologic risk factors considered include male gender, age, white race, obesity (especially central obesity), family history of BE or EAC, BE duration, endoscopic extent of BE, smoking, and gastroesophageal reflux disease (GERD) history^4^. There is a strong correlation between frequent and prolonged acid exposure and BE development; moreover patients presenting with GERD at younger ages, or with longer GERD symptom duration, are at increased risk^5^. Nevertheless, many EAC and HGD patients do not recall prior reflux symptoms. Proven endoscopic risk factors for BE progression include long BE segment length, hiatal hernia, mucosal abnormalities such as esophagitis, and the presence of BE in a 12- to 6-o’clock esophageal hemisphere^1^. Histologic factors, including intestinal metaplasia, low-grade dysplasia, and p53 overexpression are also suggested risk factors^1^.

The aims of our study are to comprehensively assess clinical, epidemiological, endoscopic, and histopathologic risk and protective factors for the progression of BE to either HGD or EAC. We reason that a clearer understanding of these factors would help optimize surveillance, since it would enable better resource allocation to surveil patients with positive, high risk factors, promoting more efficient and earlier detection of HGD and EAC.

## Methods and Materials

This study was approved by the Institutional Review Board (IRB) at the Johns Hopkins University (JHU) School of Medicine. Additionally, all methods were performed in accordance with the relevant guidelines and regulations and all informed consents (including the use of tissue samples) were obtained. All patients undergoing ≥2 upper endoscopies (EGD) ≥6 months apart, showing histologically confirmed BE from 1992–2013, were included. Consecutive BE patients with known history of BE or BE patents initially diagnosed at the start of the study were recruited as the study population. These patients were consecutive cases that were previously scheduled for surveillance endoscopy with biopsies and possible ablative therapy. We excluded patients with <2 EGDs or <6 months between initial and most recent EGDs, or EAC or HGD at initial EGD and those lack of follow up endoscopy, or insufficient records. Incident cases of HGD or EAC were identified during follow-up. Diagnosis was made by expert GI pathologists at JHU. The Center for Clinical Data Analysis (CCDA) at JHU assisted in identifying patients who underwent at least 2 EGDs with 6 months apart. If Barrett’s esophagus was noted both endoscopically (at least 1 cm of salmon colored mucosa in the tubular esophagus with intestinal metaplasia on the pathology) the patients were included as potential patients for the study. Those developing HGD or EAC were termed progressors, while those remaining free of dysplasia, developing only LGD, or regressing to normal pathology without intervention were termed non-progressors.

Electronic medical record data were collected on demographic variables such as baseline age, gender, race, and body mass index (BMI). Age was modeled as a continuous variable. Subjects fell into 3 categories of obesity: overweight (BMI 25–29.9), obese I (BMI 30–34.9), or obese II (BMI > 34.9). Smoking was categorized as never, former, or current, with former subdivided into <10, 10–30, or >30 pack-years. Alcohol use was categorized as former social, former heavy, current social, or current heavy. Illicit drug use was divided into drug type and categorized as either former or current. Caffeine intake was recorded as rare, weekly to daily, or multiple times daily. Family history of other cancers, BE, esophageal cancer, or GERD/heartburn was recorded. Medications the patient took any time after initial BE diagnosis and before diagnosis of HGD or EAC were recorded. Other epidemiologic risk factors included history of esophagitis, gastric or duodenal ulcer, gastritis, esophageal stricture, esophageal web, and Schatzki’s ring. History of GERD, heartburn, dysphagia, regurgitation, and symptom frequency were recorded, as were history of colonic adenoma, cholecystectomy, anti-reflux surgery, other cancers, hypertension, hyperlipidemia, diabetes, coronary artery disease, stroke, chronic kidney disease, and anemia. Endoscopic risk factors comprised presence and size of hiatal hernia, number and segment length of BE tongues, (long segment BE >3 cm, short segment BE <3 cm), BE circumferentially (>3 cm circumferential extent), presence of esophagitis, and presence of esophageal ulcer. Histopathologic risk factors comprised initial degree of dysplasia (low-grade or non-dysplastic).

### Statistical analyses

Demographic and clinicopathological variables were summarized with means and standard deviations for continuous variables or proportions for categorical variables. We first performed univariate logistic regression analysis with progression to EAC or the composite outcome of progression to EAC or HGD as the dependent variable and epidemiologic and clinical factors, medications and endoscopic features as independent variable. Any variable significantly associated with progression (p-value < 0.05) in the univariate analysis was highlighted as a potential risk or protective factor. We use various demographic information (such as abdominal/central obesity and BMI), past medical history (such as family history of cancer, colonic adenomas, regurgitation and anemia), medications (such as statins and SSRI), and endoscopic factors in multivariate Cox models. Multivariate Cox models used least absolute shrinkage and selection operator (LASSO) penalized regression for model selection, which minimizes the usual sum of squared errors, with a bound on the sum of the absolute values of the coefficients^6^. This method penalizes size of regression coefficients, whereby some predictors have coefficient estimates of exactly zero and can be considered “selected out” of the model. Variables with regression coefficients ≠0 were chosen for the multivariate Cox model. The tests for proportionality of hazards were done using methods as described by P. Grambsch and T. Therneau where proportional hazards tests and diagnostics were based on weighted residuals^7^. Kaplan-Meier analysis is used for progression to HGD or EAC as outcome.

## Results

**Figure 1:**
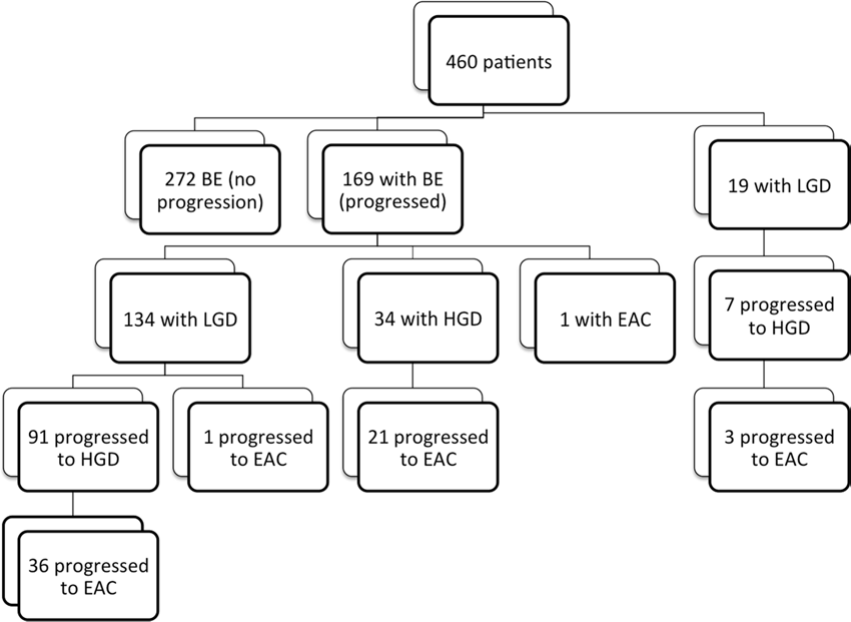
Flow-chart of patients enrolled in the study and their outcomes in regard to progression.

We identified 460 patients with BE diagnosed during the study period (see Fig. 1 for flow chart). Baseline characteristics are in Table 1 for progressors and non-progressors. We identified 272 as non-progressors and 188 as progressors. Among progressors, 19 patients were identified as low-grade dysplasia (LGD) BE and 169 patients were non-dysplastic BE (NDBE) at baseline. Average age for total cohort was 68.51 ± 13.03; 68% were males, 93% were non-Hispanic Caucasians, and 51% had smoked tobacco. Common medications were proton pump inhibitors (94%), statins (37%), aspirin (30%), angiotensin receptor blockers (ARBs, 24%), angiotensin converting enzyme (ACE) inhibitors (18%), beta-blockers (11%) and selective serotonin re-uptake inhibitors (SSRIs, 11%).

**Table 1:** Baseline characteristics of BE patients identified as progressors vs non-progressors.

	Non-Progressors (272)	Progressors (188)
**Demographics**		
**Age**	67.01 +/− 12.99	74.34 +/− 11.73
**Gender (% Male)**	174(64%)	146 (78%)
**Race**		
*Non-Hispanic Caucasian*	247 (91%)	184 (98%)
*African American*	16 (6%)	0 (0%)
*Hispanic*	3 (1%)	4 (2%)
*Asian*	6 (2%)	0 (0%)
**BMI**		
<25	62 (23%)	30 (16%)
*25–30*	100 (37%)	67 (36%)
>30	110 (41%)	91 (49%)
**Alive**	263 (97%)	178 (95%)
**Smoking History**		
*Never*	138 (51%)	84 (45%)
*Former Smoker*	100 (37%)	84 (45%)
*Current Smoker*	34 (12%)	20 (10%)
**Alcohol Use**		
*Never*	133 (49%)	72 (38%)
*Former Social Drinker*	11 (4%)	9 (5%)
*Former Heavy Drinker*	15 (5%)	12 (6%)
*Current Social Drinker*	108 (40%)	93 (49%)
*Current Heavy Drinker*	5 (2%)	2 (1%)
**Illicit Drug use**		
*Never*	253 (93%)	180 (96%)
*Former User*	8 (3%)	2 (1%)
*Current User*	11 (4%)	6 (3%)
**Family History of Cancer**	98 (36%)	96 (51%)
**Family History of BE**	5 (2%)	5 (3%)
**Family History of Esophageal Cancer**	8 (3%)	8 (4%)
**Family History of GERD**	5 (2%)	11 (6%)
**Medications**		
*NSAIDs*	35 (13%)	28 (15%)
*PPIs*	253 (93%)	184 (98%)
*H2 Blockers*	27 (10%)	7 (4%)
*Statins*	103 (38%)	66 (35%)
*Aspirin*	82 (30%)	54 (29%)
*Metformin*	22 (8%)	7 (4%)
*Oral Diabetic Medications*	8 (3%)	2 (1%)
*Insulin*	11 (4%)	13 (7%)
*ACEI*	46 (17%)	38 (20%)
*ARB*	68 (25%)	45 (24%)
*B-Blocker*	27 (10%)	23 (12%)
*CCB*	38 (14%)	34 (17%)
*Benzos*	19 (7%)	9 (5%)
*SSRI*	35 (13%)	9 (5%)
*ACEI/ARB*	73 (27%)	58 (31%)
*Calcium/Vitamin D*	56 (19%)	17 (9%)
*Diuretics*	54 (20%)	36 (19%)
**Medical History**		
*Esophagitis*	90 (33%)	60 (32%)
*Gastric Ulcer*	5 (2%)	4 (2%)
*Duodenal Ulcer*	5 (2%)	7 (4%)
*H. pylori*	8 (3%)	2 (1%)
*Gastritis*	40 (15%)	23 (12%)
*Esophageal stricture*	11 (4%)	15 (8%)
*Esophageal Web*	2 (1%)	0 (0%)
*Schatzki Ring*	16 (6%)	4 (2%)
*GERD*	266 (98%)	186 (99%)
*Colonic Adenomas*	65 (24%)	70 (37%)
*Prior Cholecystectomy*	33 (12%)	34 (18%)
*Prior Anti-Reflux Surgery*	16 (6%)	7 (4%)
*Personal History of Cancer*	35 (13%)	19 (15%)
*Hypertension*	141 (52%)	113 (60%)
*Diabetes Mellitus*	32 (12%)	24 (13%)
*Hyperlipidemia*	87 (32%)	46 (34%)
*Coronary Artery Disease*	35 (13%)	38 (20%)
*Prior stroke*	5 (2%)	5 (3%)
*Chronic Kidney Disease*	8 (3%)	5 (3%)
*Anemia*	29 (11%)	7 (4%)
*Solid Organ Transplant*	3(1%)	2 (1%)
*Caffeine Use*	112 (41%)	105 (56%)
*Heartburn*	264 (97%)	180 (96%)
*Dysphagia*	27 (10%)	38 (20%)
*Regurgitation*	33 (12%)	36 (19%)
**Endoscopic Characteristics**		
**Hiatal Hernia**	133 (49%)	100 (53%)
**Size of Hiatal Hernia**		
*Small*	76 (28%)	43 (23%)
*Medium*	22 (8%)	19 (10%)
*Large*	35 (13%)	36 (19%)
**Circumferential BE**	35 (13%)	107 (57%)
**Single BE Tongue**	256 (94%)	173 (92%)
**Multiple BE Tongues**	16 (6%)	15 (8%)
**Short Segment BE**	215 (79%)	71 (38%)
**Long Segment BE**	52 (19%)	116 (62%)
**Esophageal Ulcer Presence**	5 (2%)	4 (2%)

**Figure 2:**
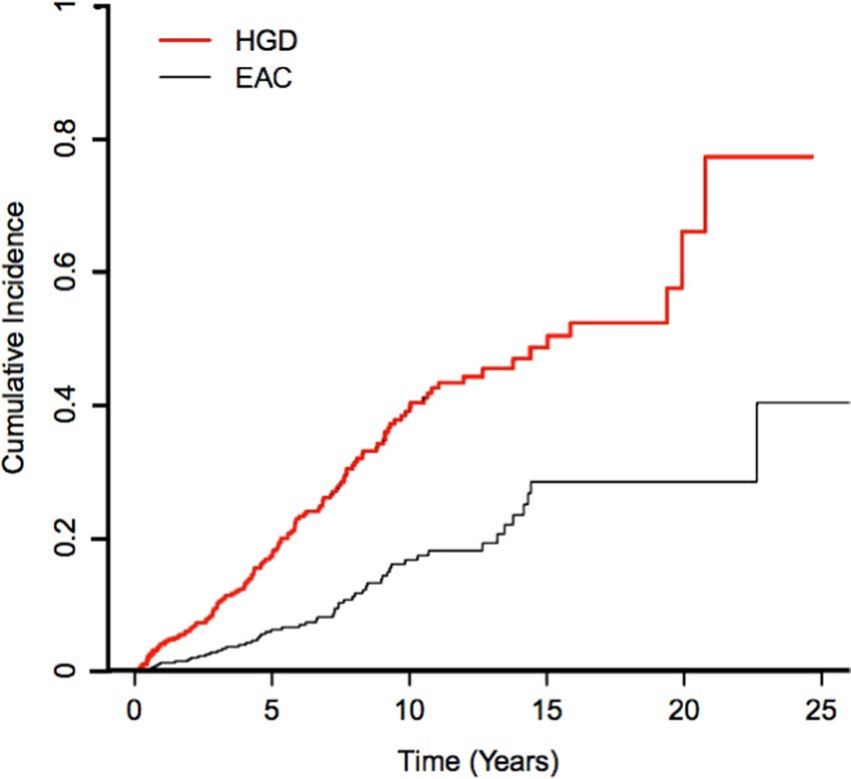
Cumulative Incidence Curves for progression to HGD and EAC for BE patients.

Of the 460 BE patients, 62 developed EAC and 132 developed HGD during the study period (Fig. 1). The mean (SD) follow-up after BE diagnosis was 7.78 (5.40) years. HGD and EAC incidences were 49.19 and 16.48 per 1,000 person-years of follow-up, respectively; 10-year cumulative HGD and EAC incidences post-BE diagnosis were 0.40 and 0.17, respectively. Cumulative HGD and EAC incidence curves are depicted in Fig. 2.

**Figure 3:**
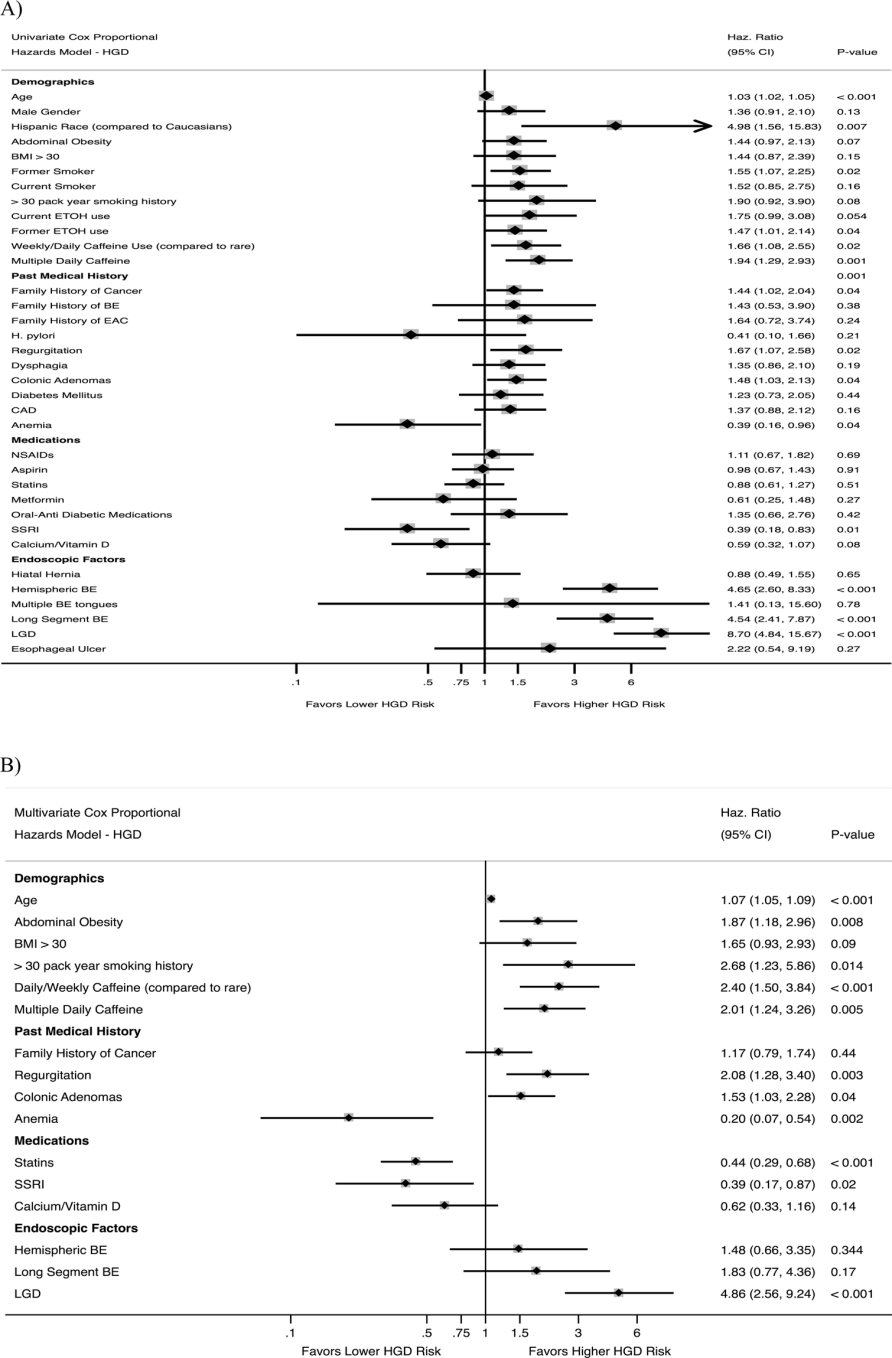
Univariate (**A**) and Multivariate (**B**) Cox Proportional Hazards Ratios for EAC.

**Figure 4:**
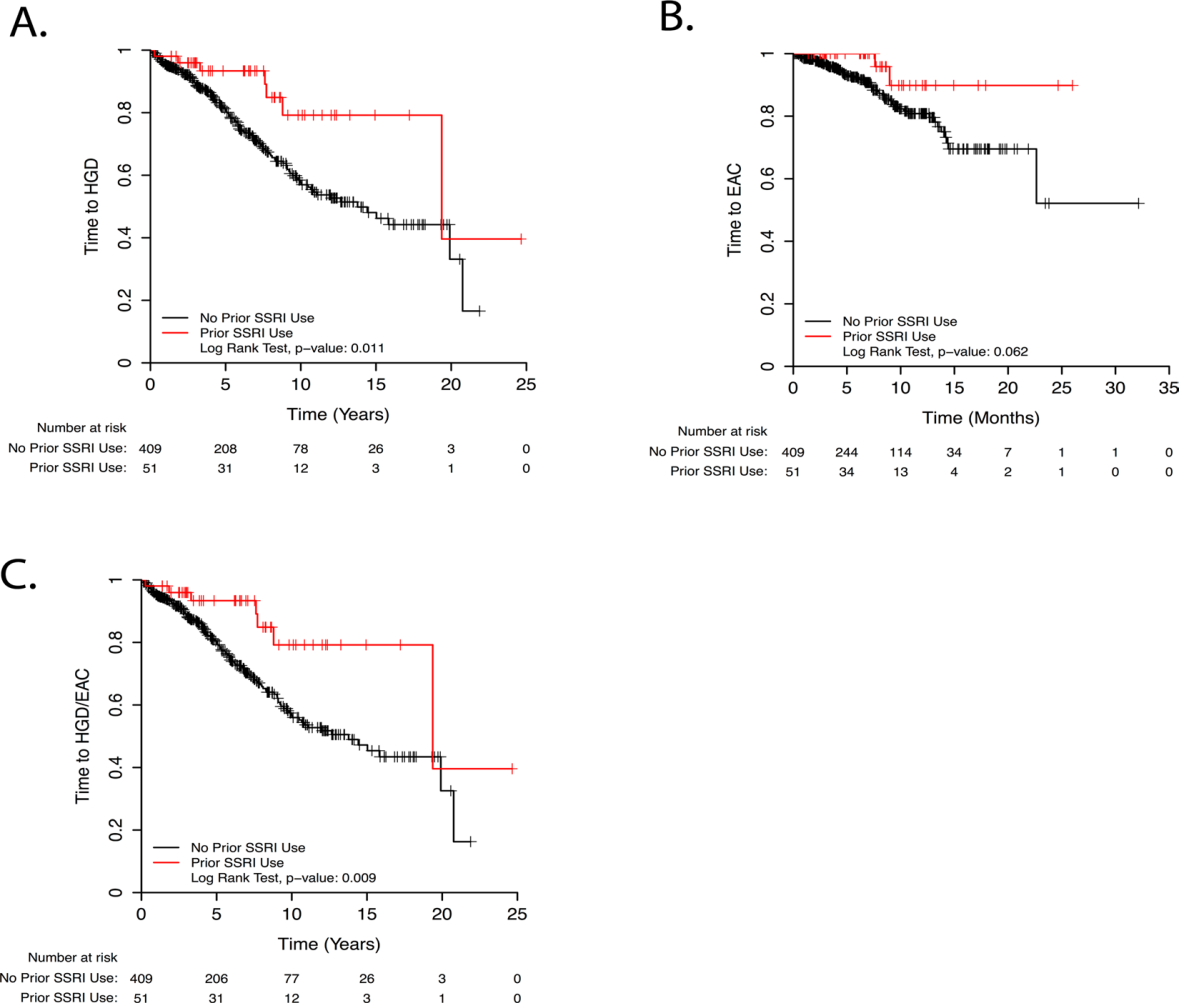
Kaplan–Meier Survival Curves for progression to HGD (**A**), EAC (**B**), and HGD/EAC (**C**) comparing SSRI users and non-SSRI users.

Results of univariate and multivariate analyses assessing the effect of various risk factors on progression to EAC are shown in Fig. 3. In univariate analysis, age, Hispanic race, abdominal obesity, history of diabetes mellitus, and oral non-metformin anti-diabetic medications were significant risk factors for progression, while SSRI usage trended strongly toward significance as protective (p = 0.08). Metformin did not significantly increase or decrease progression risk. Weekly/daily caffeine (p = 0.07), heavy smoking (p = 0.07), family history of BE (p = 0.09), and dysphagia (p = 0.09) trended positively as risk factors. Significant endoscopic risk factors were long-segment BE, presence of LGD, and development of HGD on subsequent endoscopies (patients with initial HGD were excluded). As in univariate analysis, age, abdominal obesity, weekly caffeine intake, and oral anti-diabetic medications conferred significant risk, while family history of BE trended toward significance (p = 0.10) in multivariate analysis. SSRIs were significantly protective in multivariate analysis (Fig. 4). Endoscopic risk factors in multivariate analysis included only LGD and development of HGD on subsequent endoscopies. Notably, long-segment and circumferential BE were not risk factors in multivariable analysis after adjustment for confounding factors.

**Figure 5:**
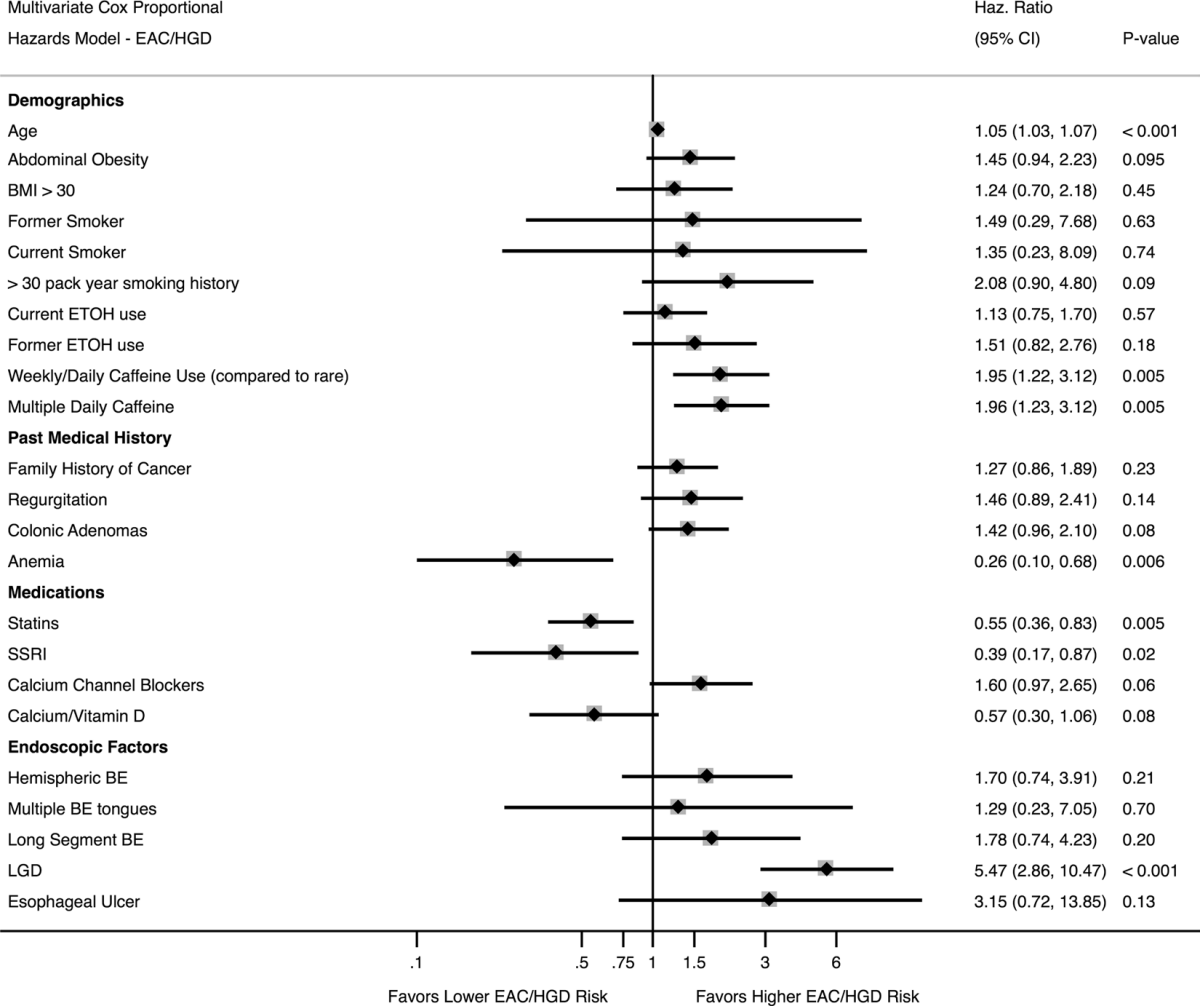
Multivariate Cox Proportional Hazards Ratio for EAC/HGD.

A multivariate model was also performed with composite outcome of progression to either HGD or EAC (Fig. 5). Significant risk factors in this model included age, weekly/daily and multiple daily caffeine usage; heavy smoking (>30 pack-years; p = 0.09), colonic adenomas (p = 0.08), and calcium channel blockers (p = 0.06) trended positively as risk factors. Anemia, statins, and SSRIs (Fig. 4c) were statistically significantly protective, while supplemental calcium/vitamin D trended towards significance as protective (p = 0.08). Significant histologic risk factors for progression included low-grade dysplasia (LGD).

## Discussion

In this retrospective study of 460 patients with histologic BE, we applied multivariate regression model to identify clinical, epidemiologic, endoscopic, and histologic risk factors for progression BE to HGD or EAC. Our results validated known risk factors for progression, including age, abdominal obesity, and smoking, but also demonstrated, to our knowledge for the first time, that caffeine intake and colonic adenomas increase progression risk. While dysplasia increased progression risk, previously reported endoscopic factors - circumferential BE, long-segment BE, and hiatal hernia - were not significant after multivariate adjustment for potential confounders. We also demonstrated protective effects of known chemoprotective medications, particularly statins, as well as several novel medications, notably SSRIs and supplemental calcium and vitamin D.

Age constituted a strong risk factor for progression to either HGD or EAC in our study, consistent with prior studies and the increased incidence of EAC reported in the Surveillance, Epidemiology, and End Results (SEER) registry^8^. Male gender is also a known risk factor for BE progression to EAC but was not significant in our study. However, SEER indicates that the largest gender difference occurs in patients younger than 65^8^. Since our average age was 68, this may explain why our gender difference was not as large. Additionally regarding obesity, we found that central abdominal obesity was a significant risk factor for both HGD and EAC, but not increased BMI, suggesting that fat distribution primarily contributed to this risk. BMI is an imprecise proxy for total body fat and was not a significant risk factor for BE in our study, which is consistent with previous studies. It should be noted that central abdominal obesity patients may have different/unhealthy life style such as tobacco and alcohol use which indirectly lead to increased risk of BE progression. The plausible mechanism for central obesity and its contribution to BE progression is that visceral abdominal obesity presses the stomach and increases intragastric pressure. This increased intragastric pressure causes a frequent reflux of gastric acid and reflux, leading to BE development. Tobacco usage is an established risk factor for BE progression, with conflicting data on caffeine usage. We found that heavy smoking (>30 pack-years) increased the composite risk of HGD or EAC by 111%. Our study also found that caffeine was a significant risk factor for the composite outcome of HGD or EAC. This is consistent with the finding that caffeine induces gastric acid secretion, relaxes the LES, and worsens GERD^9^. However, multiple studies on caffeine have yielded mixed results, with one study finding an inverse association with coffee consumption and EAC rates, while another involving 400,000 participants did not^10,11^. Only 1 study addressed BE progression risk *vs*. coffee/tea consumption: it did not find any association^12^. Our data showed that coffee and tea or caffeinated soft drink consumption, which had not been studied significantly, increased progression risk.

Colonic adenomas trended toward significance for progression to either HGD or EAC in our study. Previous studies have shown that patients with colonic adenomas have a higher risk of BE, and patients with BE have a higher risk of colonic adenomas^13^. However, to our knowledge, no studies looked at the relationship between colonic adenomas and BE progression risk. Several potential reasons may explain why colonic adenomas increase BE progression risk. First, they constitute a pre-malignant lesion with low-grade dysplasia and may represent a field defect evincing a genetic predisposition to the development of dysplasia. Indeed, inducible nitric oxide synthase and cyclooxygenase-2 are mediators of inflammation, regulators of cell growth, and elevated in colonic adenomas, colonic adenocarcinomas, BE, and EAC^14^. Additionally, 17p and 5q allelic losses are associated with progression of both colonic adenomas and BE^15^. These common genetic alterations may explain why patients with colonic adenomas have a higher risk of BE progression.

We found anemia to be potentially protective against progression to either HGD or EAC. One explanation is that anemic patients (who often undergo EGD for evaluation of anemia) represent a distinct population with a lower risk of BE progression *vs*. other BE patients, many of whom have longstanding GERD and additional risk factors and thus are screened for BE. Indeed, 2 studies in patients undergoing colonoscopy for colon cancer screening showed high rates (11% and 6.8%) of BE in asymptomatic patients^16^. These studies also demonstrated that asymptomatic patients had a lower risk of long-segment BE, previously associated with progression. Thus, anemia *per se* may not protective, but patients found to have BE without GERD or other risk factors may not be at the same risk of progression to HGD/EAC.

Previously identified endoscopic factors for BE progression include circumferential or long-segment BE^17^ and LGD^3^ or HGD^18^. In our study, long-segment BE and circumferential BE were significant only in univariate analysis but not after adjustment for other factors, divergent from prior studies^17^. Indeed, 69 different demographic, clinical, medication-related and endoscopic risk factors were measured in our multivariate analysis. Patients with long-segment BE or circumferential BE may also have concomitant risk factors increasing their progression risk. LGD was a significant risk factor to the composite outcome of HGD or EAC, and development of HGD during the study period was also a significant risk factor for progression to EAC, consistent with previous findings^19^. These findings may also explain why current endoscopic surveillance has not reduced the incidence of EAC^20^. Indeed, a recent meta-analysis demonstrated that 25% of patients with BE or BE with LGD developed an incident cancer within 1 year of endoscopic screening, questioning the ability of endoscopy to appropriately risk-stratify patients^21^. Another meta-analysis demonstrated that only 20% of EACs in BE patients were diagnosed via surveillance, whereas most EACs were prevalent, detected shortly after BE diagnosis and before potential intervention^22^. This finding, combined with our study, suggests that the priority for screening is early dysplasia detection, rather than other endoscopic findings such as length or circumferential extent, since dysplasia confers the greatest progression risk. This argues that better methods/technologies facilitating early dysplasia detection are needed to improve detection of HGD early and intervention before EAC development.

Chemoprevention of cancer is a worthy goal, particularly in BE, given the increasing incidence of EAC and the widespread prevalence of GERD and BE. Multiple studies have shown that statins reduce neoplastic progression in BE, with proposed mechanisms including anti-proliferative, anti-angiogenic, and pro-apoptotic effects^1^. In our study, statins reduced the risk of progression to the composite outcome of HGD or EAC by 48% (OR = 0.52, 95% CI = 0.34–0.79, p = 0.002). NSAIDs and aspirin have also been shown in multiple studies to be protective, but were not in our study^23^. The reasons for this include that our patient population underreported the usage of NSAIDs and/or aspirin and lack of accessible medication records (including missing cross-checked from pharmacy records) at the beginning of the study in early decades of 90 s and early 2000’s.

Two protective medications discovered by us were SSRIs and supplemental calcium or vitamin D. SSRIs have not been previously associated with a reduction in BE progression or EAC incidence. However, they exert anti-tumor effects relevant to colonic neoplasia. Specifically, SSRIs decrease cultured human colon cancer cell viability; suppress cell division in rat colonic tumors, and slow human colorectal tumor xenograft growth^24^. Additionally, SSRIs reduce growth hormone and insulin-like growth factor (IGF) levels, and IGF participates in progression of BE to EAC, based on immunohistochemical analysis of human BE, LGD, HGD and EAC^25^. Either of these mechanisms could potentially account for the protective effect of SSRIs. Vitamin D receptor expression is upregulated in BE, indicating a potential mechanism for the protective effect of vitamin D^26^.

Our study possesses several strengths. We investigated a large cohort of patients previously diagnosed with BE over a 21-year period, some of whom developed EAC or HGD during follow-up. This study comprehensively examined clinical, epidemiological, endoscopic, and histopathological risk factors. We documented not only basic clinical information, but also all medications taken, prior medical history, family history, endoscopy reports, and pathology findings. Many prior studies assessing risk factors were not so comprehensive. We also used multivariate regression modeling to limit confounding factors, unlike most published studies. Additionally, we included large sub-cohorts of patients with HGD (132 patients) or EAC (62 patients), allowing us to explore differences between progressors and non-progressors.

One limitation of our study was its retrospective nature and its restriction to one academic center. Also, some bias may have occurred due to the tertiary nature of our center and the fact that many higher-risk patients were referred from other centers because of advanced endoscopic therapies available at our hospital. Thus, 29.4% of patients with BE in our study progressed to develop HGD or EAC, a rate higher than the general BE population. Therefore, one could argue this poses a threat to external validity. However, we should be aware that our study period includes the period of 1990’s where low-resolution endoscopes were used; thus, subtle abnormalities or early neoplastic lesions in the BE segment could have easily been missed. This could lead to inclusion of patients with prevalent advanced neoplasia at baseline that was missed on the initial endoscopy, explaining for the high progression rate among patients with NDBE.

In conclusion, this retrospective study not only validates several previously identified risk factors for BE progression, including age, heavy smoking, abdominal obesity, long-segment BE, and dysplasia, but it also identifies novel risk factors, including colonic adenomas and caffeine intake. We also identified statins, SSRIs, and supplemental calcium and vitamin D as potential protective agents, each with its own biologically plausible mechanisms. These medications merit further exploration in prospective studies, along with risk factors we identified, to improve identifying patients at greatest progression risk and thus likely to benefit from more frequent endoscopic surveillance and/or earlier endoscopic therapy.
